# Positive Fluid Balance Is Associated with Higher Mortality and Prolonged Mechanical Ventilation in Pediatric Patients with Acute Lung Injury

**DOI:** 10.1155/2011/854142

**Published:** 2011-05-29

**Authors:** Heidi R. Flori, Gwynne Church, Kathleen D. Liu, Ginny Gildengorin, Michael A. Matthay

**Affiliations:** ^1^Division of Pediatric Critical Care, Children's Hospital and Research Center Oakland, 747 52nd Street, Oakland, CA 94609, USA; ^2^Division of Pediatric Pulmonology, UCSF Children's Hospital, San Francisco, CA 94143, USA; ^3^Division of Nephrology, UCSF Medical Center, San Francisco, CA 94143, USA; ^4^Department of Epidemiology and Biostatistics, CTSI, Children's Hospital and Research Center Oakland, 747 52nd Street, Oakland, CA 94609, USA; ^5^Department of Anesthesia and Critical Care and CVRI, UCSF Medical Center, San Francisco, CA 94143, USA

## Abstract

*Introduction*. We analyzed a database of 320 pediatric patients with acute lung injury (ALI), to test the hypothesis that positive fluid balance is associated with worse clinical outcomes in children with ALI. *Methods*. This is a post-hoc analysis of previously collected data. Cumulative fluid balance was analyzed in ml per kilogram per day for the first 72 hours after ALI while in the PICU. The primary outcome was mortality; the secondary outcome was ventilator-free days. *Results*. Positive fluid balance (in increments of 10 mL/kg/24 h) was associated with a significant increase in both mortality and prolonged duration of mechanical ventilation, independent of the presence of multiple organ system failure and the extent of oxygenation defect. These relationships remained unchanged when the subgroup of patients with septic shock (*n* = 39) were excluded. *Conclusions*. Persistently positive fluid balance may be deleterious to pediatric patients with ALI. A confirmatory, prospective randomized controlled trial of fluid management in pediatric patients with ALI is warranted.

## 1. Introduction

large observational studies and *post hoc* analyses of randomized trials have found that positive fluid balance during the first days after development of ALI/ARDS is independently associated with a higher risk of death [1–4]. Smaller trials have indicated that a reduction in extravascular lung water correlates with improved clinical outcomes [5–7]. In 2006, the ARDS Network published results of their Fluid and Catheter Treatment Trial [8]. This trial demonstrated that a conservative fluid management strategy, aimed at achieving normal intravascular filling pressures after resolution of shock and resulting in relative even fluid balance over the first 7 days of the study, resulted in a shorter duration of mechanical ventilation and ICU stay. 

Similar studies have not been conducted in children with ALI. The Pediatric Acute Lung Injury and Sepsis Investigators (PALISIs) network published a *post hoc* analysis of their multicenter weaning modes of ventilation study [9]. This analysis indicated that positive fluid balance was not associated with prolonged need for mechanical ventilation in children intubated for acute respiratory failure of all etiologies, but not specifically children with ALI, who were a subset of this study population.

We conducted a prospective, observational study of 320 children admitted to two large pediatric intensive care units (PICUs) that met the American European Consensus Conference (AECC) definition of ALI [10]. We hypothesized that positive fluid balance would be associated with worse clinical outcomes in children with ALI. We conducted the following *post hoc* analysis on data from our original cohort to test this hypothesis. 

## 2. Materials and Methods

For the parent investigation all pediatric patients admitted to the PICU at Children's Hospital and Research Center Oakland (CHRCO) between July 1996 and May 2000 and the University of California Medical Center, San Francisco Children's Hospital (UCSF) between July 1996 and July 1998 were prospectively evaluated. All patients who met the AECC definition of ALI [11] were included in the analyses. All patients had at least one arterial blood gas supporting the PaO2/FiO2 <300 requirement obtained as part of routine clinical care. Patients were excluded if they were <36 weeks corrected gestational age or >18 years of age, had evidence of left atrial hypertension either clinically or by echocardiogram, or had any echocardiographic evidence of intracardiac shunt. The study was approved by the Institutional Review Board at CHRCO and the Committee on Human Research at UCSF.

For the current investigation, all patients who received exchange transfusions and those patients requiring extracorporeal membrane oxygenation, continuous venovenous hemofiltration, or exchange transfusions were excluded as the total fluid balance for these patients would skew the results. All clinical data including fluid administration and losses were included in the database *a priori.*

The primary outcome was PICU mortality. The secondary outcome was ventilator-free days, defined, as in previous studies [8, 10], as the number of days the patient was alive and not mechanically ventilated in the 28 days after the onset of ALI. All patients who died while still mechanically ventilated were assigned a value of zero; all patients not requiring mechanical ventilation were assigned a value of 28. 

Data were first entered into a Microsoft Access relational database for cleaning and preparation for analysis. All clinically and statistically significant covariates from the parent investigation were included for analysis in the current investigation. Potential covariates were age, gender, ethnicity, diagnosis associated with ALI, past medical history, individual organ system failures, blood gas variables, presence of neutropenia, presence of airleak, and respiratory indices [10]. 

Cumulative fluid balance data were measured as a continuous variable over the first 3 days after onset of ALI. Daily fluid balance can be confounded by those patients that do not spend 24 hours per day in the PICU, particularly on the day of admission or in the event that the patient dies or is discharged before 72 hours. To account for this potential discrepancy, fluid balance data were recorded as mL per kilogram per hour. During data analysis, the ml per kilogram per hour variable was adjusted to 10 mL/kg/day increments to facilitate clinical interpretation of the results. 

Initial bivariate analyses included chi-square tests for categorical data and *t*-tests for continuous variables. A multivariate logistic regression model was created using a method of forward selection to examine the associations with mortality. In addition, a linear regression model was developed to determine the associations with number of ventilator-free days. The coefficient of determination *R* ^2^ was computed for each model with the Hosmer-Lemeshow goodness-of-fit test computed for the logistic model. A significance level of 0.05 was used for all statistical tests. Analyses were completed using Stata6 statistical software (StataCorp, College Station, TX, USA). 

## 3. Results and Discussion

Table 1 describes the baseline characteristics of the subgroup of 313 patients included in this analysis. As expected, these characteristics were essentially the same as the cohort examined in the parent study.

**Figure 1:**
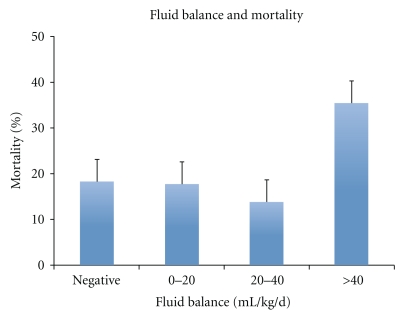
Bar graph depicting the association between cumulative fluid balance within the first 72 hours after ALI and all-cause mortality.

**Figure 2:**
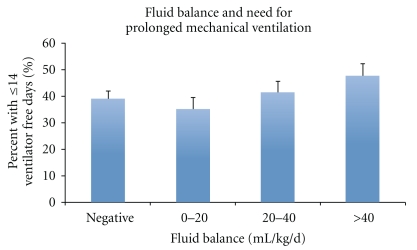
Bar graph depicting the association between cumulative fluid balance within the first 72 hours after ALI and need for prolonged mechanical ventilation (patients requiring mechanical ventilation for ≥14 days).

Bivariate logistic regression analysis demonstrated that increasing fluid balance, in 10 mL/kg/day increments, was associated with increasing mortality (OR 1.12, 95% CI 1.06, 1.20, *P* < .001, Figure 1: Chi-squared statistic, *P* < .01). Bivariate linear regression analysis also identified that an increase in fluid balance, again in 10 mL/kg/day increments, was associated with fewer ventilator-free days (Coef: −0.41, 95% CI: −0.60, −0.21, *P* < .01). To better represent this bivariate relationship graphically, this outcome was categorized to reflect patients requiring prolonged mechanical ventilation, defined as 14 or more days of mechanical ventilation (Figure 2, Chi-squared statistic, *P* = .47). 

In multivariate analysis, increasing fluid balance was associated with increased mortality (OR 1.08, 95% CI 1.01–1.15, *P* = .02, Hosmer-Lemeshow goodness of fit *P* = .87, Table 2). This effect was independent of the severity of oxygenation defect, as measured by PaO2/FiO2, at the onset of ALI and the presence of multiple, nonpulmonary and CNS organ system failures. Similarly, increasing fluid balance was also associated with fewer ventilator-free days independent of nonpulmonary organ system failure (Table 2). There was no evidence of interaction between fluid balance and cardiovascular failure, renal failure, age, or gender.

In a sensitivity analysis, multivariate models were repeated excluding the 39 patients with ALI as a result of sepsis. The association between increasing fluid balance and increased mortality and fewer ventilator-free days was similar to the primary analyses; see Table 3.

### 3.1. Discussion

This study represents the first large analysis of the possible association between cumulative fluid balance in children with ALI and increasing mortality and duration of mechanical ventilation. Although this is a *post hoc* analysis, all patients were identified prospectively and all fluid data were collected *a priori* as a part of the original study. These data are particularly relevant since there have been no large, multicenter trials testing liberal versus conservative fluid strategy in the treatment of pediatric ALI to date, though a large multicenter clinical trial of a conservative fluid management strategy in adults has been associated with an increased number of ventilator-free days and a trend towards decreased mortality [8]. 

In our original study, we concluded that there was remarkable similarity in the epidemiology and clinical risk factors associated with the clinical outcome of adults and children with ALI [10]. Biological marker studies completed in both our laboratory [12, 13] and others [14, 15] also indicate that the pathophysiology of inflammatory injury to the alveolar epithelium and lung endothelium damage associated with early ALI is similar in adults and children. 

Optimal fluid balance in the patient with ALI/ARDS is a dynamic process. The early phase of ALI is characterized by increased capillary permeability and the need to maintain intravascular volume to preserve cardiac function, in particular in patients with underlying sepsis. Fluid resuscitation may be needed to maintain intravascular volume; this may worsen underlying alveolar edema and further impair gas exchange. The question for clinicians is at what point the fluid resuscitation becomes excessive. The nonsurvivors in this study received over two times the amount of fluid per kg per day as the survivors. Were the nonsurvivors destined from the beginning to have a worse outcome based on their presenting illness, regardless of how much fluid they received? However, positive fluid balance was found to be associated with mortality independent of the severity of illness, as measured by organ system failures. This suggests that fluid overload itself may be a risk factor for mortality, regardless of the initial presenting severity of illness. 

With this as background, is this study sufficient to warrant a change in practice to that which is currently recommended in adults? We believe that the answer is not yet. Although some interventions that benefit adults with ALI are likely to benefit children with ALI, these hypotheses must be formally tested through randomized clinical trials first.

What next? One possibility is to complete similar analyses in other recently completed randomized controlled trials. Those analyses would also be subjected to the same limitations as all *post hoc*, observational analyses do, in addition to the possibility of including fewer patients than in this study. A second option would be to complete a prospective, multicenter observational study of fluid management practices similar to the PALIVE epidemiologic study of mechanical ventilation practices in pediatric ALI [16]. However, these are all observational approaches, which are subject to confounding.

Because pediatric ALI patients have a 3- to 10-fold higher mortality than the average PICU patient, there is ultimately a need for rigorously performed, randomized controlled trials of both new and more routine management practices. Ongoing collaborations between the PALISI and NHLBI ARDS Networks are specifically focusing on testing potential new ALI treatment targets, in *both* children and adults using similar algorithms and Bayesian statistical modeling to minimize the number of children that need to be enrolled. Careful consideration of study design and clinical endpoints is paramount in order to allow for multicenter, randomized, controlled trials to complete enrollment while equipoise and rigor can be maintained.

The major limitation of this study is that it is a *post hoc* analysis of a prospective study. Further, patient enrollment in the parent investigation ended in 2000 and may not reflect the full impact of lung protective mechanical ventilation algorithms. Nonetheless, the mean exhaled tidal volume in our patients was lower than the relatively injurious limb of the adult ARMA trial (10 mL/kg in this study, 12 mL/kg in ARMA). Another limitation regards the quantification of severity of illness. At the time our study was designed, the PRISM III [17] score had not yet been validated nor widely implemented for clinical use. Further, the score is only validated for the first 12 to 24 hours after PICU admission and not for the first day of diagnosis of ALI, when our data were collected. In our cohort, approximately 30% of patients developed ALI after their first day of ICU admission. Therefore, organ system failure was used to measure the severity of illness, according to current practice at that time. Central venous pressure data and serum albumin levels were not recorded in the original dataset and therefore not reflected in these analyses. Lastly, although we tested for interactions between fluid balance and cardiovascular failure (*n* = 133 patients) and/or renal failure (*n* = 47 patients) and found none, it is possible that interactions do exist within subgroups of these patients. 

## 4. Conclusion

Based on the results of recent adult trials and our observational data, we believe that positive fluid balance in the wake of ALI may be deleterious in children as it is in adults. There is a definite need to test liberal versus conservative fluid management in a large, prospective, multicenter pediatric ALI cohort. 

## Figures and Tables

**Table 1:** Patient clinical characteristics and outcomes, (*N* = 313).

Clinical characteristic	
Age, median (25–75% interquartile range)	3.4 yrs (1d–18 yrs)
Male,% (*n*)	56% (176)
PaO2/FiO2 at ALI onset^#^	161 ± 74
Diagnoses associated with ALI, % (*n*)	
Pneumonia	36% (112)
Aspiration	16% (50)
Sepsis	12% (39)
Near drowning	9% (28)
Cardiac*	7% (22)
Other	20% (62)
Nonpulmonary organ system failures at ALI onset	1.4 ± 1.5
PRISM III at ALI onset^#^	10.3 ± 8.7
Exhaled TV (cc/kg IBW)^#^	10.1 ± 4.3 cc/kg
Ventilator free days	14.8 ± 10.6 d
Mortality, % (*n*)	21% (67)

^#^Mean ± standard deviation.

*Patients with any intracardiac shunts and/or left sided heart failure excluded.

**Table:** (a) Multivariate results for mortality

	Odds ratio (95% C.I.)	*P*-Value
PaO2/FiO2^1^	0.91 (0.82, 1.00)	.05
Other OSF^2^	1.90 (1.45, 2.49)	<.01
CNS Failure	7.46 (3.60, 15.45)	<.01
Fluid Balance^3^	1.08 (1.01, 1.15)	.02

**Table:** (b) Multivariate results for ventilator-free days

	Coefficient (95% C.I.)	*P*-Value
PaO2/FiO2^1^	0.43 (0.14, 0.72)	<.01
Other OSF^2^	−2.21 (−3.07, −1.35)	<.01
CNS Failure	−6.33 (−9.06, −3.61)	<.01
Fluid Balance^3^	−0.21 (−0.39, −0.04)	.02

^1^PaO2/FiO2 measured in 20 point increases.

^2^Nonpulmonary, non-CNS organ system failure.

^3^Fluid Balance measured in 10 mL/kg/day increments.

**Table:** (a) Multivariate results for mortality, excluding patients with sepsis

	Odds ratio (95% C.I.)	*P*-Value
PaO2/FiO2^1^	0.88 (0.79, 0.99)	.03
Other OSF^2^	2.28 (1.60, 3.26)	<.01
CNS Failure	6.81 (2.94, 15.79)	<.01
Fluid Balance^3^	1.09 (1.00, 1.18)	.05

**Table:** (b) Multivariate results for ventilator-free days, excluding patients with sepsis

	Coefficient (95% C.I.)	*P*-Value
PaO2/FiO2^1^	0.41 (0.11, 0.70)	<.01
Other OSF^2^	−2.92 (−3.98, −1.87)	<.01
CNS failure	−5.56 (−8.55, −2.57)	<.01
Fluid balance^3^	−0.21 (−0.42, −0.01)	.04

^1^PaO2/FiO2 measured in 20 point increases.

^2^Nonpulmonary, non-CNS organ system failure.

^3^Fluid Balance measured in 10 mL/kg/day increment.
